# The Suitability of Trabecular Patterns in the Assessment of Dental Implant Osseointegration Process through 2D Digital and 3D CBCT Radiographs

**DOI:** 10.1055/s-0043-1772570

**Published:** 2023-09-20

**Authors:** Annisa Putri, Farina Pramanik, Azhari Azhari

**Affiliations:** 1 Department of Dentomaxillofacial Radiology Residency Program, Faculty of Dentistry, Padjadjaran University, Bandung, West Java, Indonesia

**Keywords:** cone-beam computed tomography, dental implant, osseointegration, two-dimensional radiograph

## Abstract

**Objective** The research aims to determine the suitability of the trabecular pattern in the assessment of the dental implant osseointegration process through two-dimensional (2D) digital and three-dimensional (3D) cone-beam computed tomography (CBCT) radiographs.

**Materials and Methods** This is a correlation description that explains the relationship between variables. The population consisted of 24 data points on 3D CBCT and 2D digital radiographs from the procedure after dental implants were inserted into the tibia of a New Zealand white rabbit ( *Oryctolagus cuniculus* ) on days 3, 14, and 28. The radiograph was selected based on the region of interest (ROI), which covers the peri-implant area with a width of 1 mm and length following the height of the implant. The ROI was analyzed for trabecular thickness (Tb.Th), separation (Tb.Sp), number (Tb.N), and fractal dimension.

**Statistical Analysis** The intraclass correlation coefficient (ICC) was used to statistically test the data to assess the consistency of intraobserver measurements and the *r* value (Pearson's correlation coefficient). This determines the correlation between trabecular patterns in both radiographic modalities and the Bland–Altman plot to observe the limits of acceptable discrepancies.

**Results** The ICC test showed high intraobserver consistency in trabecular pattern measurements on 2D digital radiographs and 3D CBCT. The trabecular space pattern and number showed an *r* value of 0.88 with radiographic modalities of 0.72 mm and 0.018, respectively. Additionally, the trabecular thickness and fractal dimension had an insignificant correlation, with an *r* value of 0.22, and the mean of the 2D radiograph was lower than that of CBCT.

**Conclusion** The 2D radiograph and 3D CBCT showed correlations in the trabecular number and space results but had no correlation in the trabecular thickness and fractal dimension results. Based on intraclass correlation analysis, 3D CBCT appeared to be more reliable for measuring trabecular patterns (Tb.Th, Tb.Sp, Tb.N, and fractal dimension) than 2D radiograph.

## Introduction

Osseointegration is a healing process that fixes alloplastic material to the bone without clinical abnormalities during functional loading. 1 The criteria for this process include primary and secondary stability. 2 Primary stability describes the mechanical relationship between the implant and the cortical bone to ensure that no connective tissue is formed and that healing is effective. 2 Secondary stability is a biological relationship as well as a continuation of primary stability, and it involves the regeneration of new bone remodeling around the implant. 2 3 The assessments of implant stability to bone include insertion torque technique, Periotest device, and resonance frequency analysis (RFA), although they are not recommended due to low sensitivity, high subjectivity, and lack of actual clinical condition description. 4 5 In addition, radiographic imaging is a noninvasive method used for postimplant follow-up. 4 5

Two-dimensional (2D) radiographs used in assessing bone quality are restricted to examining the degree of trabeculation (sparse to dense), although they are biased and cause discrepancy between clinicians. 6 This radiograph produces overlapping buccal and lingual bony anatomic features, grayscale variations, and inconsistent pixel values, hence it is considered less representative of good bone microarchitecture. 7 8

Three-dimensional (3D) cone-beam computed tomography (CBCT) radiography has high-resolution image scanning technology, minimal distortion, accurate linear assessment, and a significant correlation with micro-CT histomorphometry. 9 10 The drawback of 3D CBCT is tissue acquisition with implant material as well as artifact noise such as metal striking or glowing object shadows. 11 Current CBCT devices have filter enhancement to reduce this effect while the implant fluorescence is present, which may lower the diagnostic value. Meanwhile, the advantage of 2D radiographs is not causing noise on X-ray exposure with metallic materials. 11

In addition, routine 2D radiographs are preferred after dental implant insertion owing to their low cost and dose. CBCT is an enhanced radiograph that is accessible at advanced health care facilities; hence, its use as a routine follow-up is limited. 6 11 In addition, the choice of photo technique should be based on the purpose of the radiograph, availability of facilities, and patient consent. 12 13 Radiographic examination after dental implant insertion evaluates the marginal bone level and presence of peri-implantitis. 12 13 14 15 According to a meta-analysis, 2D radiographs and 3D CBCT have a high degree of compatibility for detecting marginal bone loss. 15 In another study, CBCT and 2D radiographs yielded linear measurements with the same accuracy for the detection of alveolar crest reduction between 3 and 6 mm. 12 This was in line with the publication of a systematic review in 2018, where CBCT and 2D showed a tendency to positively correlate results in viewing mesial-distal bone defects, although less significant in evaluating buccolingual intrabony defects. These studies reported that CBCT and 2D radiography are clinically acceptable and accurate for the evaluation of peri-implantitis. 16

Currently, published research is qualitatively focused on postimplant evaluation by comparing lucency levels visually for the presence of peri-implantitis and bone defects. 12 13 14 15 However, quantitative assessment of osseointegration by analyzing changes in bone microarchitecture is lacking. Based on Brånemark's observations, the creation and maintenance of osseointegration depends on the understanding of the tissue's healing, repair, and remodeling capacities. 17 18 Bone microarchitecture evaluation in dental implant treatment predicts bone strength to biomechanical loads during functional loading and mastication force, integration failure, and bone response to the healing process. 4 5 19 Bone morphometric parameters are used to observe the changes in bone structure in more detail to describe the changes in the osseointegration process. 20

Bone quantity and quality are essential factors for successful dental implantation. 18 The parameters commonly used to describe the bone microarchitecture of an implant are trabecular volume fraction (Tb.BV/Tb.TV), trabecular surface density (Tb.BS/Tb.TV), trabecular thickness (Tb.Th), trabecular separation/space (Tb.Sp), trabecular number (Tb.N), trabecular pattern factor (Tb.Pf), structural model index (SMI), connectivity density (Conn.Dn), and total porosity percentage (P(tot)). 21 The trabecular thickness (Tb.Th), separation (Tb.Sp), and number (Tb.N) are the minimum parameters that must be reported for trabecular regions. 22 Moreover, fractal dimension measurement was used to identify the bone trabecular pattern linked to bone quality, and trabecular structural changes can be detected. 4 6 19 23 According to the American Society of Bone and Mineral Metabolism and Parfitt's system, these parameters are the result of the stereology model of the primary bone index (bone volume over total volume or BV/TV). 24 This shows that the trabecular bone is composed of marrow space and plates or trabeculae with variations in thickness, distance, number, and branches. 25

The measurement of the trabecular pattern on the radiograph is expected to reveal the detailed osseointegration process. 9 23 In addition, radiographic digitization enables image processing by removing superimposed objects and separating bone marrow units from trabeculae; hence, microarchitecture quantities can be observed. 4 26 The 2D and 3D radiographs are the results of sensor recordings of objects parallel to the X-ray axis, which then produce pixel and voxel data, a combination of pixels with depth; hence, CBCT and 2D contain the same database. 7 8 Based on this phenomenon, this study aimed to evaluate the suitability of 2D radiographs and 3D CBCT for assessing trabecular pattern parameters: trabecular thickness (Tb.Th), separation (Tb.Sp), number (Tb.N), and fractal dimension in osseointegrated dental implants. The null hypothesis of this study is that 2D radiography and CBCT methods have no correlation with trabecular thickness (Tb.Th), space (Tb.Sp), number (Tb.N), and fractal dimension measurements.

## Materials and Methods

### Animal Model

This study is a correlational description to determine the relationship between two variables and uses secondary data from radiographic examination procedures based on the ethical protocol of the Animal Ethics Committee of the Faculty of Veterinary Medicine, Bogor Agricultural University, and the research permitted by the Director of Medical and Academic Services of the Oral Dental Hospital, Padjadjaran University. This procedure was performed on days 3, 14, and 28 after dental implant installation with a size of 4 × 7 mm, tapered shape, sunblasted with aluminum acid coating attached to the tibia dextra of the male New Zealand white rabbit ( *Oryctolagus cuniculus* ) with an average weight of 3 kg. In the processes of chondrogenesis, osteointegration, osteoconductive, and osteoinduction, animal models have biofunctionality relevant to human sites. 27 The rabbit tibia bone has rapid bone turnover and Haversian remodeling with a bone mineral density similar to humans and able to accommodate implant size. 28

### Trabecular Pattern Measurement

2D digital radiographs and CBCT were examined using the modalities with the equipment specifications listed in Table 1 . In the 2D examination, the object was placed parallel to the sensor with the implant perpendicular to the light source. Meanwhile, for CBCT, the object is mounted on a putty phantom model and placed on a chin support for stability and to prevent movement during exposure. The objects were positioned in the center of the field of view and longitudinally to the axis of the light source.

**Table 1:** 2D and 3D CBCT digital radiograph instrument specification

Modality	2D digital	3D CBCT
Brand	Indoray	Instrumentarium OP300 Maxio
Current (mA)	40	14
Voltage (kV)	30	90
Voxel size (mm)	**-**	0.85
Field of view (cm)	**-**	5 × 5
Exposure time (s)	00.10	15
Focal film distance	100	30
Mode	**-**	Endo Resolution
Rotation degree	Parallel to film	360 degrees

Abbreviations: 2D, two-dimensional; 3D, three-dimensional; CBCT, cone-beam computed tomography.

The inclusion criteria for the 2D digital radiographs and 3D CBCT population included complete radiographs covering all parts of the implant (coronal-apical), cortical tibia bone, and trabeculae around the implant mesial, distal, superior, and inferior; 3D CBCT radiographs with axial, sagittal, and coronal view data sets; 2D digital radiographs with anteroposterior view data set; and cranial-caudal object position. Radiographs with artifacts (nonmetal strikes) and incomplete data sets were excluded. The total sampling method was used to select 12 2D digital radiographic and 3D CBCT radiographic data sets. All radiographs were saved in standard Digital Imaging and Communications in Medicine format using the DROC software (Digital Radiology Operating Console, ECom Tech, Beijing, China) for 2D digital radiographs and the OnDemand 3D software (OnDemand 3D, KavoKerr, Daejeon, South Korea) for CBCT.

The trabecular thickness pattern, which is the thickness of each trabecular unit, was used to measure osseointegration. Trabecular separation is the distance between the units, while trabecular number is the total unit measured by the distance between the median trabeculae. Fractal dimension mathematically describes the complexity of the trabecular pattern. Also, the measurement procedure used the ImageJ software and BoneJ plugin. 29 30 This software was selected because several publications stated that it is an open-source application that does not require a special license, it is free, and has high replication and reproducibility, hence it is consistent in measuring the trabecular bone of various objects. 31

**Fig. 1:**
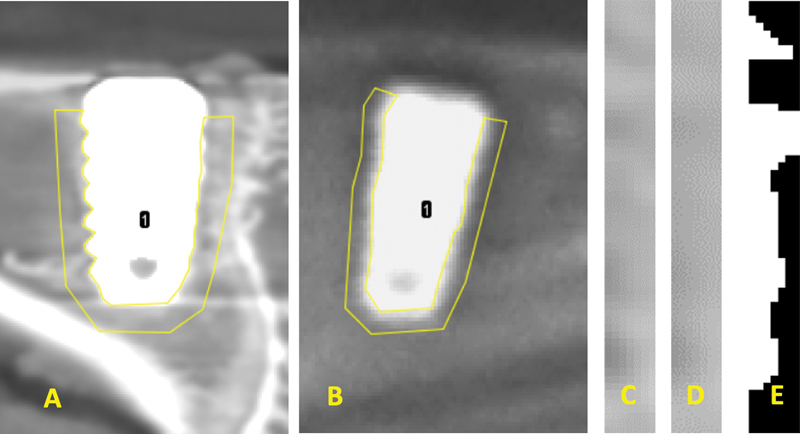
( **A** ) Region of interest (ROI) selection on cone-beam computed tomography (CBCT), ( **B** ) and two-dimensional (2D) ( **C** ) radiographs, then cropping on that section, ( **D** ) application of Gaussian blur filter, ( **E** ) and thresholding.

The radiographic analysis process initially determines the region of interest (ROI) by taking an area that includes the peri-implant and coronal-lateral bone with a width of 1 mm and a length following the height of the implant. These areas were selected based on previous publications, where they were histologically representative of the most effective osseointegration process. Huang et al studied the trabecular pattern in the lateral area, resulting in a higher pattern value than the coronal or apical area due to the variation in artifact distribution. Therefore, measuring the segments is considered to represent an osseointegrated area in this research, to avoid loss of data and reduce heterogeneity. 9 The illustration of the determination of the ROI is shown in Fig. 1 , where Fig. 1A shows CBCT and Fig. 1B shows 2D radiograph.

The image extraction was continued by cropping the selected section ( Fig. 1C ), and then a Gaussian blur filter was applied to smooth the object ( Fig. 1D ), followed by thresholding to clarify the trabecular contours ( Fig. 1E ). Subsequently, binarization separates the foreground and background, separating the trabeculae from the nontrabeculae. Trabecular pattern analysis was conducted using the tabs Tb.Th, Tb.Sp, Tb.N, and fractal dimension in the ImageJ, and numbers were generated based on the pattern. 32

### Statistical Analysis

This study (intraobserver) measured the trabecular pattern three times and continued with the intraclass correlation test to determine the consistency of the measurements. The reliability of the measurements using 2D radiography and 3D CBCT was suggested by the intraclass correlation coefficient (ICC) value. ICC value less than 0.5 indicated poor reliability, a value between 0.5 and 0.75 indicated moderate reliability, values between 0.75 and 0.9 indicated good reliability, and values greater than 0.9 indicated excellent reliability. 33 The Pearson's correlation coefficient test was used for data analysis to compare trabecular patterns on 2D and 3D radiographs. A Bland–Altman plot was used to display the data distribution to assess the limit agreement and discrepancy between the radiographs. 34 35

## Results

### Trabecular Pattern Measurement

The measurements of trabecular thickness, space, number, and fractal dimension using 3D CBCT and 2D radiography during the three phases of osseointegration (inflammation, proliferation, and remodeling) are shown in Table 2 . Based on Table 2 , the Tb.Th, Tb.N, and fractal dimension results increased in a time-dependent manner both in 3D CBCT and 2D radiographs. While Tb.Sp decreased in a time-dependent manner in 3D CBCT and 2D radiography. The Tb.Th, Tb.N, and fractal dimension measurements by 3D CBCT showed higher values than those by 2D radiographs. Meanwhile, the Tb.Sp result of 2D radiography was greater than that of 3D CBCT.

**Table 2:** Analysis of the mean and standard deviation of the trabecular pattern measurements in all the samples

Trabecula pattern	Sample	3D CBCT		2D digital	
		Mean	SD	Mean	SD
Trabecula thickness (mm)	Day 3	1.01	0.06	0.73	0.04
	Day 14	1.02	0.02	0.78	0.11
	Day 28	1.07	0.07	0.85	0.06
Trabecula space (mm)	Day 3	3.81	0.11	4.59	0.36
	Day 14	3.83	0.21	4.51	0.25
	Day 28	3.44	0.39	4.13	0.63
Trabecula number (mm)	Day 3	0.21	0.01	0.19	0.01
	Day 14	0.21	0.01	0.19	0.01
	Day 28	0.22	0.02	0.20	0.02
Fractal dimension	Day 3	2.41	0.03	1.35	0.02
	Day 14	2.41	0.03	1.26	0.14
	Day 28	2.41	0.01	1.32	0.20

Abbreviations: 2D, two-dimensional; 3D, three-dimensional; CBCT, cone-beam computed tomography; SD, standard deviation.

### The Intraclass Correlation Coefficient

The ICC values for the trabecular pattern measurements are shown in Table 3 . The measurements were performed consistently. Measurement of trabecular thickness and space using 2D radiographs showed moderate intraobserver reliability, with ICC values of 0.712 and 0.724, respectively. At the same time, the reliability of the trabecula number and fractal dimension were obtained on 2D radiographs and showed good reliability, with ICC values of 0.759 and 0.849, respectively. However, good reliability was obtained on 3D CBCT in trabecular thickness, number, and fractal dimension, with ICC values of 0.796, 0.878, and 0.797, respectively. The measurement of the trabecular space in 3D CBCT obtained a maximum index with excellent reliability, with an ICC value of 0.979.

**Table 3:** Analysis of intraclass and Pearson's correlation coefficients measuring trabecular patterns between 3D CBCT and 2D digital radiographs

	Intraclass correlation		Pearson's correlation	
	3D CBCT	2D digital	Significance two-tailed	*r*
Trabecula thickness	0.796	0.712	0.44	0.22
Trabecula space	0.979	0.724	** 0.0001 a**	**0.88**
Trabecula number	0.878	0.759	** 0.0001 a**	**0.88**
Fractal dimension	0.797	0.849	0.43	0.25

a Abbreviations: 2D, two-dimensional; 3D, three-dimensional; CBCT, cone-beam computed tomography. Intraclass correlation value < 0.5 = poor, 0.5–0.75 = moderate, 0.75–0.9 = good,  > 0.9 = excellent. A superscript (a) marks statistical significant at 0.001 significant level based on Pearson’s correlation test. The boldfaced show significant value < 0.0001.

### Pearson's Correlation Analysis

**Fig. 2:**
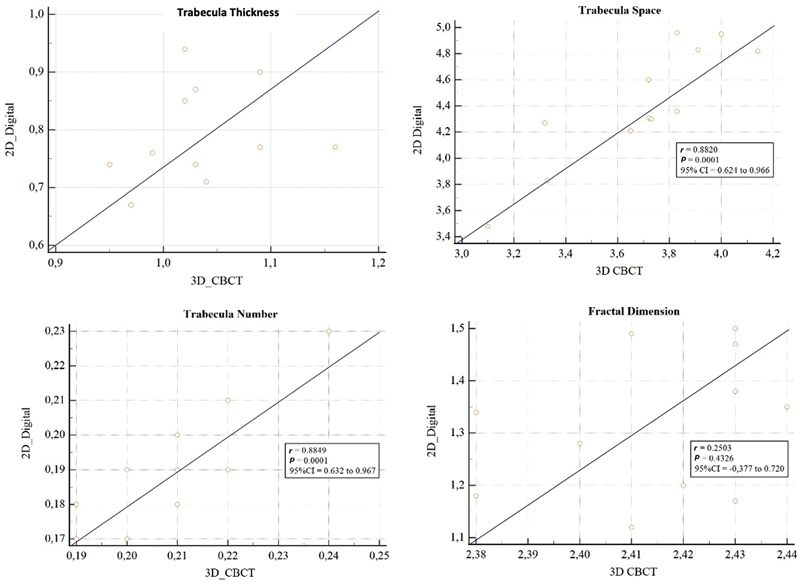
Scatter plot on each trabecular pattern.

The Pearson's correlation coefficient test was used to compare the 2D radiograph and 3D CBCT for trabecular pattern measurements, as shown in Table 3 . A scatter plot diagram of trabecular thickness, separation, number, and fractal dimensions is shown in Fig. 2 . The trabecula thickness and fractal dimension patterns obtained *r* values of 0.22 and 0.25, respectively, with a *p* -value of > 0.001. In addition, the scatter plot diagram of trabecular thickness and fractal dimension patterns shows the distribution of randomly drawn dots, indicating that there is no statistically significant correlation between 3D CBCT and 2D radiographs in the pattern. The trabecular space and number obtained an *r* value of 0.88 with a *p* -value of < 0.001, and the scatter plot diagram showed a linear pattern with a positive slope direction. This indicated that there was a statistically significant correlation between the 3D and 2D radiographs in the trabecular pattern.

### Bland–Altman Method

**Fig. 3:**
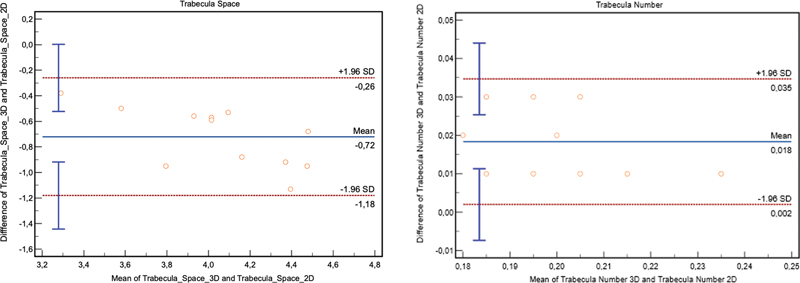
Bland–Altman curve for trabecular space and trabecular number measurement.

The Bland–Altman curve was used to describe the suitability of the space and number measurements on 3D and 2D radiographs, as shown in Fig. 3 . According to Fig. 3 , the bias value in the trabecular space, having 95% confidence interval (CI), is –0.72 mm with an agreement of –0.26 and –1.18 mm for the upper and lower limits. The trabecular number showed a bias (95% CI) of 0.018 mm ^–1^ , with an agreement of 0.002 and 0.035 mm ^–1^ for the upper and lower limits, respectively.

## Discussion

The effectiveness of dental implant treatment depends on the dynamics of anastomosis of the bone tissue with the implant surface, or osseointegration. The osseointegration process can be monitored using various methods, including radiographic analysis. In this study, the null hypothesis has been rejected because there was a correlation between the 2D radiograph and the 3D CBCT in the trabecular number and space results. 2D radiographs are still the primary method for monitoring mesiodistal marginal bone loss during postimplant follow-up. These observations have been criticized by several publications, which suggested a more specific examination by analyzing the microscopic structure in the peri-implant area. 36 37

The use of imaging software enables radiographs to reveal specific features. 36 In osseointegration, radiographic images are extracted to produce a trabecular pattern. Furthermore, the observed features included the pattern of trabecular thickness, space, number, and fractal dimension. Previous research showed that these patterns describe the microstructural bone quality and predict bone integration during the healing process. 9 10 11

This study shows that Tb.Th and Tb.N values increased while Tb.Sp value decreased considerably with time. Research on osseointegration of dental implants in canines showed that the Tb.Th and Tb.N values increased from week 2 to week 12, and Tb.Sp decreased significantly over time. 38 Moreover, a previous study by Fang et al confirms that Tb.N and Tb.Th had increased, while Tb.Sp decreased considerably with time at 1, 4, and 8 weeks after implantation. 39 This indicates that the results of this study are in accordance with those of previous studies. Tb.N and Tb.Th directly reflected the amount of new bone that had formed. 39 During osseointegration, the increase in the trabeculae thickened because of the growth of lamellar bone fibers in the implantation area. 40 New bone formation resulted in a decrease in the trabecular bony gap and the Tb.Sp value decreased with time. 39

The results showed that 3D CBCT radiographs had higher trabecular thickness and fractal dimension values than 2D radiographs. In addition, statistical tests showed that the correlation as a parameter of osseointegration assessment was very low and insignificant. This is because both modalities have different detector types, light source distances to the object, and device settings (kVp, mA, and exposure time); hence, they produce images with different pixel sizes. 41 Parsa et al, who discovered the trend of 2D radiographs underestimating 3D CBCT results, stated that low resolution causes poor contrast-to-ratio. A blur image contrast affects the software during digital processing; hence, the quality of the trabecular structure is not optimal. 42 The results of 2D measurements showed inconsistency in each sample that differs from the 3D as shown by statistical tests and scatters plots which revealed heterogeneous and random results.

The smaller the pixel size, the higher is the spatial resolution and the better is the contrast. The resolution is a pixel density that describes the level of detail in the stored image. 43 Contrast is the distribution of dark and light based on the difference between the highest and the lowest intensity values composing the pixels in the image. 43 CBCT produces smaller pixel units; hence, thin trabeculae are visible compared with 2D, which may not capture the thin portions of bone spicules. 44 In addition, variations in trabecular thickness occurred because on days 3, 7, and 28, the earliest phase of the osseointegration process, and histologically, inflammatory and granular tissue was more dominant than bone tissue.

In this study, the processing of radiographs using the ImageJ software was semiautomated. ROI was selected manually owing to variations in the dental implant position in the bone from other research radiographs. The intraclass coefficient was used to determine consistency in implementing the ROI, which ICC values of 2D radiograph and 3D CBCT were high. The segmentation process continued after converting the selected ROI into a binary image. It is fully automated at this stage, where the bone image is binary owing to the global thresholding feature; hence, the microscopic trabecular pattern is seen. 7 Waarsing et al stated that publications on radiographs with objects derived from metal using a global threshold reduce the diagnostic value. 45 This is because different ionization absorptions produce different color intensities in one image. The noisy overlapping trabeculae and the metal striking of titanium smeared out, hence the size is thinner and does not represent the actual density of the image. 45

The measurement of trabecular space and number showed a good correlation between the modalities, and the scatter plot diagram revealed that the samples were homogeneous. Meanwhile, the compressed trabecular spicules are still rounded owing to the thresholding process, although the bone structure shrinkage does not cause a significant difference. The Altman–Bland curve statistical analysis revealed that the measurement discrepancies were only 0.72 and 0.018 mm.

Specific local thresholds and paying attention to the color distribution on the histogram have been used to improve the phenomenon of global thresholding inconsistency. However, only radiographs with submillimeter resolution such as micro-CT images uses local thresholds. 46

The success of dental implants can be achieved by evaluating several implantation parameters such as bone loss, pain, mobility, prosthesis, radiolucency, surrounding soft tissue, and the patient's subjective assessment. 47 This study has some limitations. In this research, Tb.Th, Tb.Sp, Tb.N, and fractal dimension were used to assess the osseointegration process in dental implant. However, the osseointegration process can be assessed using other parameters such as trabecular volume fraction (Tb.BV/Tb.TV), trabecular surface density (Tb.BS/Tb.TV), trabecular pattern factor (Tb.Pf), SMI, connectivity density (Conn.Dn), or total porosity percentage (P(tot)). In future research, a larger sample size and more appropriate parameters can be added to verify the osseointegration process. In addition, it is necessary to test with other methods, such as RFA or histomorphometric analysis, to complete the information and clinical decisions. Our results suggest that CBCT is more reliable to assess osseointegration in dental implant than 2D radiograph. Dentists should consider using CBCT to evaluate the dental implant osseointegration process because it can determine all aspects of the treatment, from planning to surgery to final restoration.

## Conclusion

The 2D radiograph and 3D CBCT have correlation in the trabecular number and space results, and have no correlation in the trabecular thickness and fractal dimension results. The intraclass correlation analysis shows that 3D CBCT have a good reliability on Tb.Th, Tb.N, and fractal dimension and excellent reliability on Tb.Sp. The 2D radiograph has moderate reliability on Tb.Th and Tb.Sp, and good reliability on Tb.N and fractal dimensions. Based on these, 3D CBCT appeared to be more reliable for measuring trabecular patterns (Tb.Th, Tb.Sp, Tb.N, and fractal dimension) than 2D radiograph.
